# Reproductive interference and Satyrisation: mechanisms, outcomes and potential use for insect control

**DOI:** 10.1007/s10340-022-01476-6

**Published:** 2022-02-08

**Authors:** Christina Mitchell, Stewart Leigh, Luke Alphey, Wilfried Haerty, Tracey Chapman

**Affiliations:** 1 School of Biological Sciences, University of East Anglia, Norwich Research Park, Norwich, NR4 7TJ UK; 2 The Pirbright Institute, Ash Rd, Pirbright, Woking, GU24 0NF UK; 3 Evolutionary Genomics, Earlham Institute, Norwich Research Park, Norwich, NR4 7UG UK

**Keywords:** Reproductive interference, Satyr effect, Satyrisation, Interspecific interactions, Pest control, Pest management

## Abstract

Reproductive Interference occurs when interactions between individuals from different species disrupt reproductive processes, resulting in a fitness cost to one or both parties involved. It is typically observed between individuals of closely related species, often upon secondary contact. In both vertebrates and invertebrates, Reproductive Interference is frequently referred to as ‘Satyrisation’. It can manifest in various ways, ranging from blocking or reducing the efficacy of mating signals, through to negative effects of heterospecific copulations and the production of sterile or infertile hybrid offspring. The negative fitness effects of Satyrisation in reciprocal matings between species are often asymmetric and it is this aspect, which is most relevant to, and can offer utility in, pest management. In this review, we focus on Satyrisation and outline the mechanisms through which it can operate. We illustrate this by using test cases, and we consider the underlying reasons why the reproductive interactions that comprise Satyrisation occur. We synthesise the key factors affecting the expression of Satyrisation and explore how they have potential utility in developing new routes for the management and control of harmful insects. We consider how Satyrisation might interact with other control mechanisms, and conclude by outlining a framework for its use in control, highlighting some of the important next steps**.**

## Key Messages

- Reviews the current knowledge on interspecific mating interactions.
- Synthesises factors that impact frequency or asymmetry of Reproductive Interference.
- Analyses of implications/outcomes of interacting factors of Reproductive Interference with test cases.
- Generates framework for using fitness cost asymmetries for pest control.

## Introduction

The study of the rapid evolution of reproductive traits and their divergence between closely related species is of fundamental interest to researchers in the context of speciation. It also gives insights into introgression and biodiversity conservation (Pfennig and Pfennig 2010; Rice and Pfennig 2010; Shuker and Burdfield-Steel 2017). There is empirical evidence that the divergence of different reproductive traits between closely related species, whether morphological or behavioural, can occur at variable rates. This can result in the phenomenon whereby individuals from the diverging species cannot form fertile hybrids, but can suffer negative fitness costs due to interspecific sexual interactions. These reproductive interactions can take various forms and are collectively referred to as ‘Reproductive Interference’. In vertebrates and invertebrates, this process is often termed Satyrisation (after the sexually promiscuous half-goat man of Greco-Roman myth; Ribeiro and Spielman 1986; Bargielowski et al. 2013). The effects and fitness costs of reciprocal matings between species are often asymmetric, and it is this aspect that has implications for species coexistence (Gröning and Hochkirch 2008; Shuker and Burdfield-Steel 2017; Kyogoku 2020), as well as pest management. Some authors originally used the term Satyrisation to refer exclusively to *asymmetric* Reproductive Interference. However, the usage of this term has since broadened, and in this review, we define Satyrisation as symmetric or asymmetric Reproductive Interference that occurs in vertebrate and invertebrate mating systems.

Reproductive Interference sits at the interface between evolutionary biology and ecology. For instance, there is a growing realisation that it can help to resolve unexplained features of competitive relationships between species, such as when species exclusion cannot be explained by resource competition (Park et al. 1948; Birch et al. 1951; Kishi et al. 2009). There is also a growing awareness that Reproductive Interference can be a driver of reproductive character displacement, in addition to reinforcement and the Templeton effect (Templeton 1981; Butlin and Ritchie 2009; Hollander et al. 2018).

Reproductive Interference is also relevant for conservationists, as it could influence the invasion success of non-native species, and result in impacts upon other species with which the invasives could potentially interbreed (Liu et al. 2007; Gröning and Hochkirch 2008; D’Amore et al. 2009). Reproductive Interference is of significant applied interest in terms of its potential utility in controlling harmful species including disease vectors such as *Aedes* mosquitoes (Gröning and Hochkirch 2008; Bargielowski and Lounibos 2016). Satyrisation is being considered as a potential pest control method, both independently and in conjunction with other current pest-suppression strategies (Leftwich et al. 2016; Honma et al. 2019).

The first aim of this review is to summarise the ways in which Satyrisation is expressed within vertebrate and invertebrate systems and to determine the factors that result in asymmetric fitness costs, using illustrative test cases. The second aim is to consider how the principles underlying Satyrisation could be deployed for the control and management of dangerous insect pests. To do this, we reviewed the current literature on Satyrisation, defining the factors that cause its effects to vary, and used this to inform how it can be deployed directly or indirectly as a method of pest control.

We restrict this review to the consideration of situations in which any hybrid progeny that are produced from matings between species have zero fitness (i.e., they are inviable or sterile). The topics of hybrid matings leading to introgression and hybrid vigour are covered in detail elsewhere (Huxel 1999; Hill et al. 2020) and are not considered within the scope of this review.

## Reproductive interference

Reproductive Interference is a broad term that is used to define the situation when there are sexual/reproductive interactions, usually between individuals of closely related species, which do not lead to the production of fertile hybrids and instead result in negative fitness costs for the interacting individual males and/or females (Gröning and Hochkirch 2008; Shuker and Burdfield-Steel 2017; Kyogoku 2020). This can include interactions between even reasonably diverged species, such as when a territorial male seeks to exclude individuals of other species, as well as its own, during mating competitions. In this way, Reproductive Interference can represent a potential intersection between resource competition and heterospecific (between different species) rivalry (Drury et al. 2015). However, Reproductive Interference more often occurs between species which are closely related/ recently diverged, due to the existence of incomplete mating barriers. Therefore, Reproductive Interference is fundamentally linked to reproductive character displacement, reinforcement and speciation (Smadja and Ganem 2005; Kronforst et al. 2007; Matute 2014). It can occur, in principle, over a broad range of plant and animal taxa (Levin 1970). The study of Reproductive Interference, to date, has been focussed mostly on the study of plant science (Weber and Strauss 2016). In particular, the emphasis has been on determining the mechanisms and origin of Reproductive Interference in the formation of post-zygotic barriers leading to speciation. Asymmetries in Reproductive Interference in plants have also been reported in terms of unilateral incompatibility (Bedinger et al. 2011; Lewis and Crowe 1958; Marta et al. 2004) and vestigial viable pollen (Whitton, et al. 2017). These factors are known to limit species co-occurrence.

In vertebrate and invertebrate mating systems, reproducing individuals are usually mobile and may exhibit a complex range of reproductive behaviours. This has the potential to offer a greater number of scenarios in which Reproductive Interference might occur, in comparison to plants, and to lead to stronger selection to avoid costly interspecific coupling (Levin 1970). Reproductive Interference that occurs within animal mating systems is usually referred to as Satyrisation, and is divided into seven categories (Gröning and Hochkirch 2008; Shuker and Burdfield-Steel 2017) each related to distinct types of mating barrier. These mechanisms can be pre- or post-copulatory, often work in conjunction, and can have potentially different ecological impacts. The mechanisms are: signal jamming, heterospecific rivalry, misdirected courtship, heterospecific mating attempts, erroneous female choice, heterospecific mating, and hybridization (summarised, with examples, in Table 1).

**Table 1:** Summary of different Satyrisation categories (Gröning and Hochkirch 2008) together with illustrative examples

Type of reproductive interference	Form of incomplete mating barrier	Description	Examples
Signal jamming/Signal Interference	Pre-mating	Disruption of reproductive signals due to presence of heterospecific signals such as pheromones, mating calls, visual displays	Responses to heterospecific pheromones in Lepidopterans (moths) (Landolt and Heath 1987)
Heterospecific rivalry	Pre-mating	Heterospecifics are mistaken for conspecific rivals and become subject to aggression	Interspecies fighting and territoriality in *Hetaerina* damselflies (Drury et al. 2015)
Misdirected courtship	Pre-mating	Courtship of heterospecifics due to mistaken identity/similarity of courtship behaviours and responses	Courtship of heterospecific females by males of butterfly species *Leptidea sinapis* and *Leptidea juvernica* (Friberg et al. 2013)
Heterospecific mating attempts	Pre-mating	Incomplete heterospecific copulations, which can have fitness costs arising from bodily harm, or harassment effects	Attempted forced copulation between male guppies (*Poecilia reticulata*) with heterospecific female Topminnows (*Skiffia bilineata*) (Valero et al. 2008)
Erroneous Female Choice	Pre-mating	Females actively choose heterospecific males due to mistaken identity or pre-existing sensory bias	Erroneous female choice in *Paratrechalea ornata* spiders*,* in which females accept nuptial gifts and engage in misdirected courtship with male *Paratrechalea azul* (Costa-Schmidt, and Machado 2012)
Heterospecific mating	Post-mating, pre-zygotic	Successful heterospecific coupling, where fitness costs can arise from bodily harm, gamete wastage, and the induction of refractoriness to further matings	Heterospecific mating and insemination between male *Aedes albopictus* and female *Aedes aegypti* mosquitoes (Nasci et al. 1989)
Hybridization	Post-mating, post-zygotic	Production of zero fitness hybrid offspring, from heterospecific mating. Fitness costs depend on extent of energetic costs expended on the production of hybrid offspring	Production of sterile hybrids in *Drosophila arawakhana* × *Drosophila dunni* crosses. (Hill et al. 2020)

Reproductive Interference shares some features of resource competition and is density-dependent (Gröning and Hochkirch 2008). For example, it can result in population or species exclusion, or coexistence through divergence (Kuno 1992). This has been modelled using a Lotka-Volterra competition framework (Ribeiro and Spielman 1986; Kuno, 1992). As with competition, Reproductive Interference can result in either exclusion of the ‘weaker’ species, divergence (parapatry), or coexistence through niche partitioning/reproductive character displacement/ eventual evolution of complete mating barriers (Kyogoku 2020). However, unlike resource competition, Reproductive Interference lacks a true ‘shared resource’, and instead occurs due to errors in, or incomplete, mate recognition, resulting in fitness reductions in individuals of the interacting species (Gröning and Hochkirch 2008).

Due to the shared features of Reproductive Interference and resource competition, it can often be difficult to disentangle the relative importance of these different forms of interspecific interactions on reproductive behaviours, particularly within field settings. However, there is a growing realisation that Reproductive Interference may play a larger role in species competition and speciation than previously considered (Hochkirch et al. 2007). For example, it may help to explain the results of experiments initially attributed to competitive exclusion in which the seemingly weaker resource competitor excluded the ‘more efficient’ species (Park et al. 1948; Birch et al. 1951; Kishi et al. 2009). Reproductive Interference may even be maintained in some cases due to what Drury et al. (2019) refer to as an ‘Evolutionary Catch-22’, wherein the cost to males of mating with heterospecific females is lower than that of missing conspecific (between same species) mating opportunities, thereby limiting divergence in male mate recognition and female reproductive characteristics (Shuker and Burdfield-Steel 2017). Whilst this is unlikely to be a feature of all species that can experience Reproductive Interference due to differences in male fitness costs, it is nevertheless interesting to consider in the context of factors that may limit the evolution of reproductive character displacement (Drury et al. 2015, 2019). Overall, our knowledge of Reproductive Interference is important in the context of how we consider species interactions and their possible ecological outcomes. This is particularly relevant to increased invasion events, in which consideration must be given to the effects of Reproductive Interference on invasion success and how it impacts upon introgression into at-risk species (Liu et al, 2007; Gröning and Hochkirch 2008; D’amore et al. 2009).

## Asymmetric reproductive interference/Satyrisation

An intriguing aspect of Reproductive Interference is the high degree of asymmetry in fitness costs often observed in reciprocal interactions between species (Gröning and Hochkirch 2008). This can rapidly increase the probability or rate of competitive exclusion or niche partitioning. Within invertebrates, Satyrisation is beginning to garner attention as a potential mechanism for intentional exclusion to achieve pest control (Leftwich et al. 2016; Honma et al. 2019). The term ‘Satyr’ was first utilised in this context by Ribeiro and Spielman (1986) and was originally defined as *asymmetric* Reproductive Interference by reference to a mathematical model that explored the fitness costs of reciprocal interspecific interactions. However, since then, Satyrisation has generally been used to describe the symmetric *and* asymmetric Reproductive Interference that occurs in vertebrates and invertebrates, and this is the definition we adopt here. An example of asymmetric Satyrisation can be found in cryptic butterfly species, where the less competitive and less reproductively efficient species are observed to exhibit rapid niche partitioning with respect to their more competitive counterparts. This is thought to arise at least partly to avoid costly misdirected courtships from heterospecific males (Dincă et al. 2013; Friberg et al. 2013). Satyrisation was first described several decades ago (e.g., Ribeiro and Spielman 1986; Miller et al.^1994^) and interest in it is growing partly as it provides an explanation for the observed reduction of *Aedes aegypti* populations in North America (particularly in the panhandle of Florida) following the invasion of *A. albopictus* (Parker et al. 2019). Satyrisation has been thoroughly studied in the *Aedes* system, in both laboratory and field experiments (Nasci et al. 1989; Tripet et al. 2011; Carrasquilla and Lounibos, 2015; Bargielowski and Lounibos, 2016; Honório et al. 2018; Bargielowski et al. 2019). This has led researchers to evaluate how prevalent it might be in nature, and to explore methods to utilise its principles to reduce or exclude pest species in favour of more benign ones (Honma et al. 2019). The main applied focus on Satyrisation stems from the finding that it often has asymmetric effects on fitness. The fitness costs to females engaging in courtship with heterospecifics are typically higher than the costs for males of heterospecific interactions, due to the generally higher levels of reproductive investment made by females. This scenario sets up the risk of energetic costs due to gamete wastage, potential harm from mating with males with incompatible morphology or physiology, or opportunity costs of lost mating opportunities due to the induction of post-mating refractoriness (Bath et al. 2012; Bargielowski and Lounibos, 2016; Yassin and David, 2016; Leigh et al. 2020). An example is described by Tripet et al. (2011) in which low (0.01–12.3%) mating rates to conspecifics were observed in female *A. aegypti* following injection with *A. albopictus* male accessory gland extracts, which induce refractoriness to remating in both species. Failure to mate with a conspecific will incur significant fitness costs. Asymmetry in costs in reciprocal interactions between species pairs is also common, with, for instance, females of one species suffering much higher costs heterospecific interactions than the other. Tripet et al. (2011) provide evidence, by observing that *A. aegypti* females are rendered refractory to mating by the heterospecific male accessory gland proteins of *A. albopictus*, whereas the insemination of *A. albopictus* females by *A. aegypti* male accessory gland proteins has no such effect.

Differential rates of character divergence and the underlying drivers are key candidates for producing asymmetric effects of Satyrisation. Studying the mechanisms of these asymmetries could also yield important insights into the relative plasticity or conservation of genes that regulate sexual behaviour and physiology and the rate at which they evolve, as well as strengthening our overall understanding of reproductive isolation. Asymmetric Satyrisation could also potentially inform new methods of control by the repression or replacement of pest species, in a manner that could bypass restrictions and concerns associated with genetic modification (Alphey et al. 2013; Leftwich et al. 2016; Honma et al. 2019). The effects of Satyrisation within existing control programmes are also of potential significance. For example, Satyrisation between modified males released to effect control with heterospecifics resident in the target control area (e.g., release males courting heterospecific non-target females, or heterospecific males blocking matings for release males) might reduce the efficacy of control, by lowering the frequency of conspecific matings between released males and wild females.

Research into Satyrisation, as a direct method of pest control, is still in its infancy. However, its potential to interfere with key reproductive processes means that knowledge of the fundamental mechanisms involved could indicate new routes for manipulating pest populations into increased vulnerability. A key aspect is to understand which factors most influence asymmetric fitness costs between species. In addition, it will be important to determine if control could be rendered more successful by simultaneously manipulating multiple factors that increase Satyrisation asymmetry, or by tailoring the approach to target asymmetries to which any specific target population is particularly vulnerable. The factors of greatest importance in determining overall levels of Satyrisation are likely to vary with control scenarios and are discussed in more detail below.

## Factors impacting the degree of asymmetry in Satyrisation

The efficacy of Satyrisation at driving species exclusion (whether via sexual exclusion or a combination of sexual and competitive exclusion) or niche partitioning, is highly dependent on the degree of asymmetry in fitness costs between the interacting species (Ribeiro and Spielman 1986). The asymmetry is strongly influenced by a variety of factors including density dependence, evolutionary history, and life history trade-offs. These factors and their effects are illustrated in Table 2.

**Table 2:** Factors that affect the degree of asymmetry in Satyrisation

Factors influencing the extent of asymmetry in Satyrisation	Consequences of factors
Relative abundance, population density, and sex ratio of target species and satyr species upon introduction	Affects the frequency of heterospecific interactions and matings
Pre-existing asymmetry in resource competition	Can exacerbate population dynamics that influence reproductive interference and increase the likelihood of exclusion
Number of generations spent in sympatry or allopatry	Influences degree of selection pressure to prevent interspecific reproductive interactions
Presence/degree of pre-mating barriers	Mate recognition, choosiness, phenology of mating, courtship differences can alter asymmetry of fecundity costs of hybrid mating between species
Presence/degree of post-mating barriers	Effectiveness of responses to heterospecific seminal fluid proteins, the extent of con or heterospecific sperm precedence, refractory period, and capacity to hybridise can all alter asymmetry of fecundity costs of hybrid mating between species
Degree of intraspecific sexual conflict within the target species and satyr species	Can influence asymmetry of heterospecific mating fitness costs
Fitness costs of Satyrisation resistance genes	Influences likelihood of resistance evolution/how long it takes for resistance to evolve/how long resistance genes will stay in the population if the species become allopatric
Life History trade-offs: parasite load, predation, changes in fecundity over time, life history, etc	General fitness effects that can influence relative abundance and fecundity
Mating system	Differences in mating system will result in species differing in pre-mating and post-mating investment
Presence of multiple interbreeding species	Could alter relative fitness costs between species and change selection pressures

There is an inherent difficulty in disentangling the relationships between Satyrisation and species character traits in order to ascertain whether an existing character trait simply exacerbates Satyrisation, or if Satyrisation itself was, or is, a driver of trait evolution. For example, we need to understand whether resource competition simply intensifies the effects of Satyrisation or if individuals of the less competitive species will be selected to specialise to avoid Satyrisation, as is suggested to occur in conflicts between the ladybirds *Harmonia axyridis* and *H. yedoensis* (Noriyuki et al. 2012).

It should also be noted that the extent of Satyrisation is also highly likely to be influenced by changes to abiotic factors and habitat structure. Examples include habitat loss or climate change potentially pushing related species together or preventing niche partitioning. This could increase the frequency at which Satyrisation occurs, by either creating sympatry where species were once allopatric (i.e., creating new habitat overlaps between species) or by increasing population densities in hybrid zones (Liu et al. 2007). Such factors may also cause changes to preferred ecological niches, which may act in conjunction with Satyrisation. The following sections discuss in more detail the various factors proposed to affect the efficacy/frequency of Satyrisation (Table 2).

### Population density/species ratio

As with resource competition, the relative abundance of each competing species will play a role in whether Satyrisation is strong enough to result in species exclusion. Under resource competition, an increased number of competitors results in resource limitation, whereas under Satyrisation, an uneven species ratio or a high density can result in a high frequency of heterospecific encounters, increasing the likelihood that costly heterospecific courtship will occur (Kyogoku and Sota, 2017; Kyogoku 2020). This phenomenon was observed in simulations by Takafuji et al. (1997) based on interactions between two closely related spider mites, in which the initial density ratios heavily affected the extent of competitive exclusion that occurred. This has significant implications for the success of invasion by non-native species which can reproductively interfere with native species.

### Pre-existing resource competition asymmetry

As Satyrisation often occurs between closely related species, resource competition may be strong as there may not yet have been sufficient divergence to avoid niche overlap. Theory by Kishi and Nakazawa (2013) predicts some of the ways in which Satyrisation and resource competition can interact. In situations where the more resource-efficient species also suffer lower fitness costs from Satyrisation, this should result in the exclusion of the weaker species being more likely or more rapid. In contrast, when fitness cost asymmetries in resource competition and heterospecific reproductive interactions occur in opposite directions, i.e. the more resource-efficient species are more negatively affected by heterospecific reproductive interactions and *vice versa*, Satyrisation could theoretically lead to species coexistence, or even favour the weaker competitor. Another example of how resource competition and Satyrisation can have a combined effect on local species exclusion is found in pied and collared flycatchers on the Swedish Island of Öland (Vallin et al. 2012). Resource competition between these two species over mating territories led to young pied flycatcher males being unable to establish territories. This in turn reduced the abundance of conspecific Pied Flycatchers males available, leading to an increase in heterospecific matings, the costly production of low-fitness hybrids, and eventual local exclusion. The excluded species was found to have partitioned across separate islands, potentially to avoid the combined effects of resource competition and Satyrisation (Vallin et al. 2012).

### Number of generations in sympatry/allopatry

Researchers investigating Satyrisation in *Aedes* have shown that mild forms of resistance to Satyrisation can evolve within just a few generations (Bargielowski et al. 2013, 2019; Bargielowski and Lounibos, 2016). However, this means that allopatric populations may often be more susceptible to Satyrisation. Bargielowski and colleagues have observed that in *A. aegypti,* resistance to Satyrisation was associated with an increased female choosiness in sympatric populations, with allopatric females showing lower levels of discrimination against heterospecifics (Bargielowski et al. 2013, 2019; Bargielowski and Lounibos, 2016). Similarly, Kyogoku (2020) observed that Satyrisation is more likely to occur during secondary contact (e.g., previously allopatric species coming into contact) than within coexisting (e.g., sympatric or parapatric) species.

### Presence/degree of pre-mating barriers

The presence, and effectiveness, of pre-mating barriers between closely related species will necessarily affect the extent and frequency with which negative fitness costs are experienced. Hence, these barriers are key to the existence and extent of Satyrisation. For example, in diverging species that retain the capacity to interbreed, one direction of the cross may often be more common than the reciprocal, due to one species having developed stronger pre-mating barriers than the other. This is likely to be dependent on the evolutionary history of divergence between species. Hence, consideration of the time since divergence and/or phylogenetic relatedness may allow researchers to estimate the accumulation of changes in reproductive characteristics (Coyne and Orr 1989), and thus, the likely strength of pre-mating barriers. An example of the evolution of pre-mating barriers that lead to fitness cost asymmetries is observed between *Drosophila occidentalis* and *D. suboccidentalis*, with *D. suboccidentalis* females being less receptive to heterospecific mating than *D. occidentalis* females*,* when measured in a series of no-choice tests (Arthur and Dyer 2015).

### Presence/degree of post-mating barriers

The completeness of post-mating, pre-zygotic mating barriers between closely related species can affect the fitness costs of Satyrisation. The magnitude of post-mating barriers will, as for pre-mating ones, depend upon the evolutionary history of divergence between the species involved. An example is found in the phenomenon of conspecific sperm-precedence, in which same species sperm are used preferentially over that of any other species sperm present in the female reproductive tract. Hence, even if heterospecific mating can be costly, the fitness costs of gamete wastage could potentially be mitigated via conspecific sperm-precedence, provided that females can or have previously mated with a conspecific male (Burdfield-Steel et al. 2015). Price (1997) and Rugman-Jones and Eady (2007) observed conspecific sperm precedence in *Drosophila simulans* and *Callosobruchus subinnotatus,* respectively, and noted that conspecific sperm was not only used preferentially for fertilisation but was also stored preferentially in spermathecae. However, it was not evident to what extent these phenomena were controlled by preferential female use, or by physiological effects of male seminal fluid proteins. A recent model by Iritani and Noriyuki (2021) of the reproductive interactions between the ladybird beetles *Harmonia axyridis* and *H. yedoensis* suggested that conspecific sperm precedence would not be sufficient to counteract the negative effects of Satyrisation. This was due to the high costs of increased refractoriness to conspecific mating following a heterospecific mating. Overall, the efficacy of post-mating barriers in reducing the costs of Satyrisation will vary between species according to the relative costs of pre- versus post-mating effects on reproductive success.

### Degree of intraspecific sexual conflict within the target species and Satyr species

Some research into Satyrisation has suggested that intraspecific sexual conflict between the evolutionary interests of each sex may play a role in explaining asymmetry in the fitness costs of hybrid matings between species (Shuker et al. 2015; Leigh et al. 2020). In species that experience high levels of sexual conflict, females may be better adapted to tolerating the aggressive actions of seminal fluid proteins or persistent courtships. Similarly, females from species subject to lower levels of sexual conflict might be ill-equipped to mitigate the coercive and harmful effects of mating with ‘harmful’ heterospecific males. Yassin and David (2016) found evidence to support this hypothesis as they observed differences in female mortality between hybrid crosses in the *Drosophila melanogaster* species subgroup. In crosses with higher mortality, females were often found to have higher levels of melanisation in their abdominal regions, suggesting wounds from heterospecific mating were more severe in some crosses than others. Similarly, Kyogoku and Sota (2015) found that exaggerated genital spines in the sexually competitive males of the seed beetle *Callosobruchus chinensis* mediated the costs of Satyrisation in *C. maculatus* females. This suggested a direct link between male-male intraspecific competition adaptations, and fitness cost asymmetries in Satyrisation.

### Fitness costs of ‘Satyrisation resistance’ genes

If Satyrisation carries high asymmetric fitness costs, it is likely to select for the evolution of resistance within the species which suffers the highest costs (Bargielowski et al. 2013, 2019). However, if the selection is relaxed, e.g., if exposure to the Satyr species is reduced, Satyrisation resistance genes may be rapidly eliminated. This has been observed by Bargielowski et al. (2019) who described a reduction in Satyrisation resistance traits in *A. aegypti* when they were no longer found in sympatry with *A. albopictus*. The fitness costs were unknown but were suggested to be related to increased female choosiness, which can act to prevent hybrid matings when both species are in sympatry but which may restrict mating opportunities with conspecifics in allopatry. The impact of costs of resistance genes is therefore important to consider, as it can influence the maintenance of resistance to Satyrisation and determine which populations will be or become more susceptible to it.

### Life history trade-offs

Factors such as predation, parasite load, and nutritional resources that influence selection pressures and life history will likely have impacts on the existence of Satyrisation, its level of asymmetry, and its effect on sexual exclusion. For example, Drury et al. (2015) considered that Satyrisation was being maintained in sympatric populations of *Hetaerina* damselflies due to weak selection pressure on male mate choice and limitations in female character displacement, as a result of the requirement to maintain crypsis and avoid predation. In addition, Bargielowski et al. (2019) observed an increase in receptivity to heterospecific mating (in *A. aegypti* ♀ x *A. albopictus* ♂ crosses) as individuals aged, likely due to a willingness in females to accept lower quality mates as age-specific fecundity decreased. This could itself have density-dependent effects, since the time to find a mate (or at least a male) is likely to increase as density decreases. We conclude that accurate determination of the occurrence and effects of Satyrisation requires consideration of demography and many different biotic interactions.

### Mating system

Mating systems are expected to have major effects on fitness costs associated with hybrid matings. For example, for the mating systems in which each reproductive episode involves a significant investment (e.g., by the giving of nuptial gifts) or in species in which there are limited reproductive opportunities, then even small differences in reproductive characteristics between species could alter the level of Satyrisation asymmetry and result in divergent fitness costs. This phenomenon is evident in interactions between different biotypes of the haplodiploid whitefly *Bemisia tabaci.* Haplodiploidy (i.e. haploid males produced from unfertilised eggs and diploid females from fertilised) renders the frequency and success of mating an important determinant of sex ratio, and thus can greatly affect population growth. It was found that between the B and Q biotypes of *B.tabaci,* the B biotype was more behaviourally plastic. When exposed to Satyrisation effects from exposure to the Q biotype, B biotype females more readily accepted copulations from B males, allowing for the maintenance of sex ratio. In contrast, Q biotype appeared invariant in their mating acceptances and did not upregulate their acceptance of con-biotype mates (Crowder et al. 2010).

#### Presence of multiple interbreeding species

The dynamics of interspecies breeding can be complex if more than one reproductively interfering species is present in sympatry. This can affect relative fitness costs depending on the frequency at which each species courts/interbreeds with others. Females could mate heterospecifically with different species, potentially on multiple occasions. Shuker et al. (2015) considered heterospecific mating and harassment between four species from the bug family Lygaeidae (*Lygaeus equestris, Spilostethus pandurus, Lygaeus creticus* and *Oncopeltus fasciatus*) and found rare but consistent patterns of heterospecific matings between all species. In mass-breeding experiments, the presence and/or identity of the companion bug sex and species had significant effects on nymph production. In no-choice mating assays, heterospecific pairings between female *L. equestris* and male *S. pandurus* resulted in a particularly large reduction in *L. equestris* female longevity and fecundity. Some of these species have overlapping distributions in nature, thus Satyrisation has the potential to occur between these species in the wild. It would be interesting to investigate such instances of Satyrisation between multiple interacting species because of the wide variety of ecological outcomes to which they could lead.

## Satyrisation as a control method

Following the observations that Satyrisation effects arising from *Aedes albopictus* were likely to have been a primary driver behind the population decrease of *Aedes aegypti* in North America (Tripet et al. 2011; Bargielowski et al. 2013, 2015) researchers have become interested in exploring the principles of Satyrisation for intentional population exclusion (Leftwich et al. 2016; Honma et al. 2019). The fact that Satyrisation occurred within *Aedes* species has been key to the increasing interest in its use for control, as decades of research have sought to discover effective methods to limit these important arbovirus vectors that spread globally significant pathogens such as dengue, chikungunya and Zika viruses (Alphey et al. 2013; World Health Organisation 2014; Parker et al. 2019).

However, despite being observed in North American *Aedes* populations, it is challenging to determine how frequently Satyrisation occurs in the field (Crowder et al. 2010; Bargielowski et al. 2015). If Satyrisation were to be used for control, the release of both sexes of the interfering species would operate via population replacement (replacing the pest with a more benign species). In contrast, the release of just one sex would function via population suppression (reducing or eliminating the pest; Alphey et al. 2013; Alphey 2014). However, it is possible that any replacement species could cause additional and potentially unanticipated problems. For example, *A. albopictus* is itself an arbovirus vector of medical significance, though it may be a less efficient vector for the transmission of relevant arboviruses than is *A. aegypti* (Alphey et al. 2013; Hugo et al. 2019). The relative vector competence of *Aedes* species is highly dependent on which disease and disease strain they carry (Vega-Rúa et al. 2014). Even if *A. albopictus* was confirmed as a less competent vector, it is not yet clear whether the release of more vectors could offset any benefit created by the reduction of original pest species.

Additional traits may also deserve consideration. For example, *A. albopictus* is reported to exhibit more aggressive biting behaviour than *A. aegypti*. Hence, the additional nuisance of releasing more biting insects into a target area for control should be assessed. For this reason, in scenarios involving disease vectors such as *Aedes* species, it is generally beneficial to release only males, as it is females that bite, require blood meals, and result in further disease transmission (Alphey et al. 2013; Gilles et al. 2014; Zhang et al. 2015). Provided that the females of the target pest show sufficient susceptibility to heterospecific courtship/mating and that this incurs sufficient fitness costs, male-only releases could be compatible with the aim of population control via Satyrisation. As a consequence, there is interest in understanding the molecular mechanisms of Satyrisation in order to engineer Satyr strains for control that could target both inter- and intraspecific reproductive interactions. For example, there is evidence to suggest that Satyrisation can occur between isolated populations within species, which are undergoing incipient speciation (Wu et al. 1995; Ting et al. 2001). Therefore, it may be possible to identify or engineer strains to confer control through within-species Satyrisation effects. This, when combined with recognition of the factors described above that increase population susceptibility to Satyrisation, could be fruitful. In effect, this could resemble control via sterile males or via the Sterile Insect Technique (SIT, or mass release of sterile males to effect population control) and would also resemble an interference control strategy originally developed in *Culex pipiens fatigans* (Krishnamurthy and Laven 1976) in which strains of the same species were available that were incompatible (though not initially known, the basis of this incompatibility was infection with different types of *Wolbachia*).

As with all control methods, Satyrisation will be susceptible to the evolution of resistance (Bargielowski et al. 2013) and being rendered less effective by the expression of sexual traits such as conspecific sperm-precedence. However, resistance genes often carry fitness costs (Crowder et al. 2010; Bargielowski et al. 2019) meaning that in the absence of any selective pressure due to the presence of ‘Satyr’ individuals, resistance should decay. This creates an opportunity to determine which pest populations are more likely to be susceptible to Satyrisation by analysing how long they have been in sympatry or allopatry.

The dependence of Satyrisation on incorrect mate choice could also create opportunities for synergies with other control methods, with the aim of inducing additive or even multiplicative effects (Leftwich et al. 2016). Analysis and alteration of the genetic qualities of a target population and release strain, such as non-target loci, could be used to complement primary control strategies (Leftwich et al. 2021). For example, one could ensure that any release strain intended to confer one primary mechanism of control, such as via *Wolbachia* infection, was also sensitive to Satyrisation. Release of such insects for control could then introgress Satyrisation sensitivity alleles into the target population simultaneously with any primary targeting genes (Alphey et al. 2009). This would create an opportunity to subsequently exploit the sensitivity to Satyrisation introgressed into the target population, to enhance the efficacy of future management.

Similarly, Honma et al. (2019) examined “Sterile Interference”, i.e., a combined application of the sterile insect technique and Satyrisation. In this, they explored how control programmes could be made cost-effective, using the initial reduction of the conspecific population to increase the ratio of heterospecific males to conspecific males, and therefore increase the likelihood of interspecific mating. Any control programmes in which engineered or manipulated individuals are released into a target population (e.g., such as Sterile Insect, or Incompatible Insect Techniques) should consider the possible effects of Satyrisation. Any appreciable frequency of courtships or matings between released individuals and heterospecifics in the area will decrease the efficacy of control by increasing mating interference and reducing the probability of the conspecific pairing upon which control is predicated.

The idea that Satyrisation may be affecting pre-existing control methods underlines that Satyrisation shares characteristics with these successful management schemes, namely the utilisation of signal jamming and mating disruption to exert control over pest populations. The potential difference between these methods may be that Satyrisation could have a greater role in affecting pre-mating fitness costs, which could be used to bolster the reproductive losses experienced by the pest population due to unsuccessful copulations. In addition, Satyrisation control programmes could reap the benefits of single-sex release, but without the potential fitness losses from treatments that induce sterility in individuals released as part of Sterile Insect Technique programmes.

Our understanding of the potential of Satyrisation as a direct method of control is as yet undeveloped. However, while this means the Satyrisation is not likely to be applied in the near future, its understanding is vital both to understand its potential impacts upon control via other mechanisms and to inform potential new routes for control. By considering Satyrisation when designing control initiatives, we can determine and anticipate its likely positive or negative impacts.

## A framework for control via Satyrisation

A potential framework for considering Satyrisation for control would require several key steps, and these are outlined below and in Table 3. Of primary importance would be to identify the target population requiring control and from this to determine (i) whether it has any closely related species with which is it shows Satyrisation, and (ii) if these species occur in sympatry. If no such examples exist, additional research would be required to determine if Satyrisation has been observed between any related species in laboratory experiments. Subsequent steps would be to consider whether it is ethical, straightforward and beneficial to potentially release the ‘controlling’ Satyr species into the area containing the target species, through a series of standard risk assessments (Touré et al. 2003; Bale et al. 2008). Analyses from previous biological control and genetic pest management schemes could be used as a foundation (FAO/IAEA 2006; Oye et al. 2014). There are clear parallels between the potential use of Satyrisation and biological control, either in its standard or augmented form (i.e. if ongoing releases are required). Whether releasing the Satyr species/population complies with this current and well-established legislation for biological control would need to be carefully assessed (Turner et al. 2018) as well as considering biosafety frameworks advised by global authorities on biosecurity and public health (WHO and UNICEF 2010; United Nations 2003; Engineering Biology Research Consortium 2020).

**Table 3:** Overview of planning elements for potential Satyrisation control protocol development and associated steps

Required plan components	Reasonable steps
Target identification and rationale	(i) Identify target species
	(ii) Identify potential "Satyr" species
	(iii) Determine the frequency of reproductive interference through observations in sympatry or laboratory experiments
Risk assessment and regulation compliance	Research local regulations on species release and control protocols. Consider ethical and ecological ramifications of control
Examine the efficacy of potential satyrisation control procedure	(i) Consider factors discussed in Table 2, and how these may affect the frequency and success of Satyrisation
	(ii) Examine potential synergies with other control methods
Consider practical applicatory elements	(i) Cost-effectiveness
	(ii) Duration, location, and frequency of application
	(iii) Communication with stakeholders and public

It would be important in this assessment to focus on elements of the process that are potentially Satyrisation-specific. These might centre around the relationship between field and laboratory studies and the potential for resistance. Satyrisation in field populations with a long history of sympatry might represent ‘resistant’ genotypes, and effective control strategies via Satyrisation in this context would be encouraging. Isolated populations of the target species may be much more susceptible to Satyrisation, and this could be revealed by laboratory studies. However, such populations could rapidly acquire resistance. From a regulatory perspective, there may be quite a difference between introducing a new species (to create Satyrisation) versus supplemental releases of one of two species already in sympatry.

If it is determined that the release of a Satyr population for control is ethical, safe, and beneficial, it will be necessary to examine how each factor function between the target and Satyr population (Table 2). This may include:

1. Conducting field cage and then open field observations of interspecific interactions, both sexual and competitive.
2. Population and demographic surveys and modelling of populations.
3. Laboratory and field cage recreations of mating assays to determine the mating frequency and to observe pre- and post-mating barriers.
4. Crossing species over multiple generations, first in the laboratory and then in semi-natural conditions, to ascertain how resistance genes arise and persist.
5. Examining the genetic and geographical history of the target population, to determine their susceptibility to Reproductive Interference.
6. Examining the degree of intraspecific sexual conflict in each species/population.

If, after examining these factors, Satyrisation remains a viable prospect, it should be considered whether it can synergise with other control methods, such as SIT (Honma et al. 2019). Following this, the development of practical control elements would be followed (Table 3) and under guidance from the various regulatory authorities (Vanderplank 1944; FAO/IAEA 2006; Bale et al. 2008; Turner et al. 2018).

Overall, considering the ever-growing problems of resistance to standard chemical pesticides, and with such pesticides often being non-specific and harming non-target species, it is important to assess all potential alternative methods for control (Alphey et al. 2013; Shelton et al. 2020). Satyrisation could easily be added to this list, as it is a naturally existing phenomenon that could be harnessed in a number of different ways. Our growing understanding of Satyrisation invasion dynamics and the potential ecological complications of species release, will aid in the future development of principles of Satyrisation as a pest control method.

## Conclusions

Satyrisation operates at the interface between evolutionary genetics and ecology and there is a growing body of literature to demonstrate its importance in the natural world via effects on species exclusion, speciation, and partitioning (Ribeiro and Spielman 1986; Kuno 1992). There are various factors that can influence the presence and degree of Satyrisation, including density-dependent factors such as species abundance, through to rates of species divergence and variation in sexual conflict. From an ecological point of view, we need to consider how Satyrisation may shape species distributions, and how it may alter invasion success and dynamics. From an evolutionary perspective, we must also consider the extent to which Satyrisation has influenced speciation and reinforcement. From an applied perspective, appropriate use of Satyrisation may aid in suppressing pathogen vector populations or increasing crop yield by limiting crop pest populations.

## Author Contributions

CM, TC and WH conceived and planned this article; CM wrote the first draft; all authors contributed to and approved the final manuscript.

## Data Availability

There are no raw data to deposit for this article.
